# Genetic and Functional Analyses of *Cutibacterium Acnes* Isolates Reveal the Association of a Linear Plasmid with Skin Inflammation

**DOI:** 10.1016/j.jid.2023.05.029

**Published:** 2023-07-20

**Authors:** Alan M. O’Neill, Kellen J. Cavagnero, Jason S. Seidman, Livia Zaramela, Yang Chen, Fengwu Li, Teruaki Nakatsuji, Joyce Y. Cheng, Yun L. Tong, Tran H. Do, Samantha L. Brinton, Tissa R. Hata, Robert L. Modlin, Richard L. Gallo

**Affiliations:** 1 Department of Dermatology, University of California San Diego, La Jolla, California, USA; 2 Department of Cellular & Molecular Medicine, University of California San Diego, La Jolla, California, USA; 3 Department of Pediatrics, School of Medicine, University of California San Diego, La Jolla, California, USA; 4 Division of Dermatology, School of Medicine, University of California Los Angeles, Los Angeles, California, USA

## Abstract

*Cutibacterium acnes* is a commensal bacterium on the skin that is generally well-tolerated, but different strain types have been hypothesized to contribute to the disease acne vulgaris. To understand how some strain types might contribute to skin inflammation, we generated a repository of *C. acnes* isolates from skin swabs of healthy subjects and subjects with acne and assessed their strain-level identity and capacity to stimulate cytokine release. Phylotype II K-type strains were more frequent on healthy and nonlesional skin of subjects with acne than those isolated from lesions. Phylotype IA-1 C-type strains were increased on lesional skin compared with those on healthy skin. The capacity to induce cytokines from cultured monocyte-derived dendritic cells was opposite to this action on sebocytes and keratinocytes and did not correlate with the strain types associated with the disease. Whole-genome sequencing revealed a linear plasmid in high-inflammatory isolates within similar strain types that had different proinflammatory responses. Single-cell RNA sequencing of mouse skin after intradermal injection showed that strains containing this plasmid induced a higher inflammatory response in dermal fibroblasts. These findings revealed that *C. acnes* strain type is insufficient to predict inflammation and that carriage of a plasmid could contribute to disease.

## INTRODUCTION

Acne vulgaris is a disease of the pilosebaceous unit and the eighth most prevalent disease worldwide, affecting 9.4% of the global population (Hay et al., 2014; Vos et al., 2012). The high disease prevalence and its associated clinical, psychosocial, and economic impact lead to a significant disease burden. *Cutibacterium acnes* is a gram-positive, facultative anaerobic bacterium that is the most abundant bacterial member of the skin microbiome, colonizing lipid-rich areas of the skin at densities of up to 10^6^ colony-forming units (CFUs)/cm^2^ (Leyden et al., 1998). Despite its abundance on normal skin, *C. acnes* is considered a major contributor to the development of acne and the host inflammatory response. Furthermore, despite the presence of *C. acnes* in all skin follicles, only a small proportion of follicles exhibit the inflammation characteristic of acne, even in severe cases (Ramli et al., 2012). Thus, the bacterial–host pathogenic determinants that regulate these irregular inflammatory events in acne remain incompletely understood.

The precise role of *C. acnes* in the etiology of acne vulgaris has remained elusive for decades owing to similar bacterial CFU counts in follicles from patients with acne and healthy individuals (Puhvel et al., 1975). More recent analysis of the acne skin microbiome confirmed similar relative abundances of *C. acnes* in the follicles isolated from acne and healthy skin (Fitz-Gibbon et al., 2013). However, more detailed analyses revealed differences in the *C. acnes* strain populations between patients with acne and those without acne. *C. acnes* strains found on acne skin most often belong to phylotype IA-1 (representing ribotypes RT4, RT5, and RT8), whereas health-associated strains belong to other phylotypes of IA-2, IB, or II (representing RT1, RT2, RT3, and RT6) (Barnard et al., 2016; Fitz-Gibbon et al. 2013). Since then, other studies have adopted high-resolution sequencing approaches to type *C. acnes* strains and have generally confirmed the finding that phylotype IA-1 is more abundant on subjects with acne (Conwill et al., 2022; Hall et al., 2018). Furthermore, some strains showed a higher potential to induce cytokines from cultured monocytes (Yu et al., 2016). These observations have led to the hypothesis that *C. acnes* phylotypes have a role in disease development.

In this study, we sought to better understand the factors that predict *C. acnes* inflammatory potential. To do this, we collected clinical *C. acnes* isolates from lesional and nonlesional skin of patients with acne as well as from the skin of healthy volunteers and screened this repository for the capacity to induce cytokines and induce inflammation when injected into mice. We report that *C. acnes* strain types associated with acne are found on acne lesional skin but not on nonlesional skin and that inflammatory potential is not exclusively associated with specific strain types. By combining whole-genome sequencing with functional assessment of *C. acnes* isolates, we observed that the presence of a linear plasmid was a predictor of inflammatory potential. These observations provide a greater understanding of how *C. acnes* contributes to inflammation in acne.

## RESULTS

### Epithelial inflammatory responses to health- and disease-associated *C. acnes* strains are cell-type dependent

**Figure 1.**
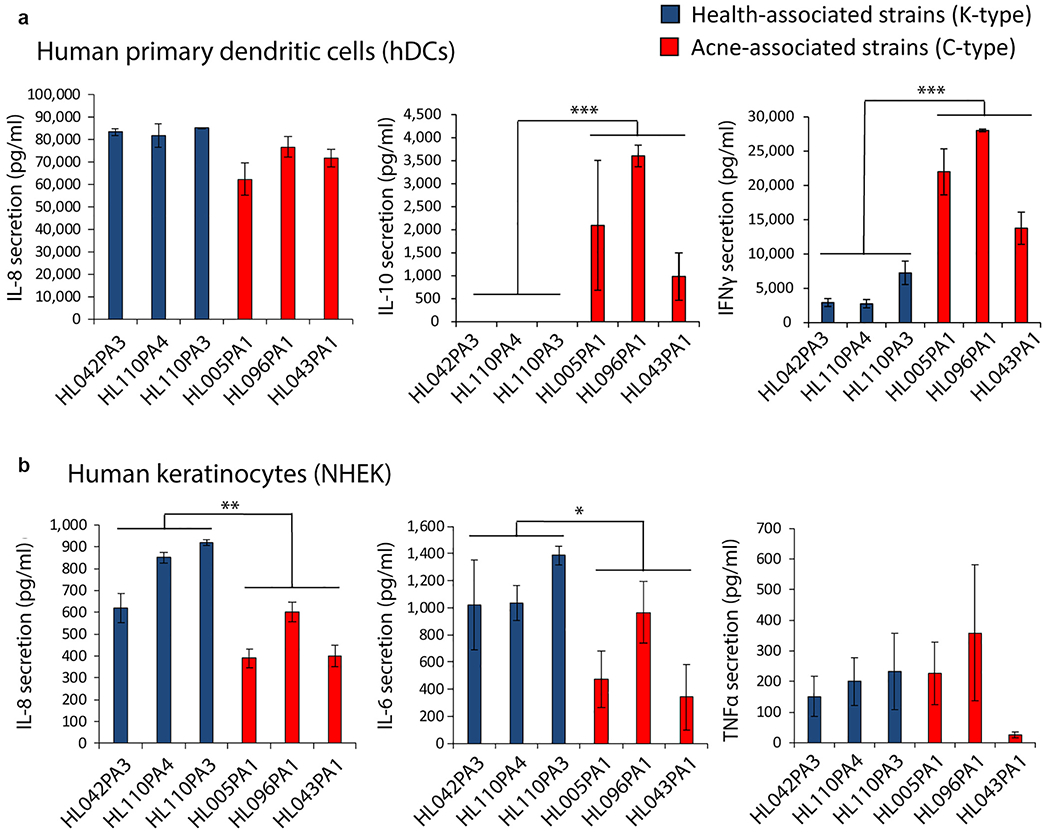
Different inflammatory capacities of *C. acnes* strains based on cell type. (**a, b**) Quantification of secreted proinflammatory cytokines by ELISA from (**a**) primary hDCs and (**b**) NHEKs 24 h after treatment with 15% sterile conditioned media from health-associated *C. acnes* strains (blue) and acne-associated *C. acnes* strains (red). Data shown indicate mean ± SEM. n = 3; *P < 0.05, **P < 0.01, and ***P < 0.001. h, hour; hDC, human monocyte-derived dendritic cell; NHEK, normal human epidermal keratinocyte.

To investigate the host response to *C. acnes*, we compared the response of different host cells to six health- and acne-associated strains. *C. acnes* isolates were cultured in nutrient and lipid-rich, anoxic conditions for 5 days and then sterile filtered, and this conditioned media was added onto the culture of host cells for 24 hours. In agreement with published literature (Nagy et al., 2005; Yu et al., 2016), acne-derived strains (C-type strains HL096PA1, HL043PA1, and HL005PA1) induced greater levels of IL-10 and IFN-γ from human monocyte-derived dendritic cells than health-associated K-type strains HL110PA3, HL110PA4, and HL042PA3 (Figure 1a). However, the opposite relationship was observed upon exposure of normal human epidermal keratinocytes (NHEKs). These host cells had less IL-8 and IL-6 release after exposure to acne-associated C-type strains than to the health-associated K-type strains (Figure 1b). In addition, cytokine levels from NHEKs or human monocyte-derived dendritic cells were similar upon treatment with conditioned supernatant of all three strains belonging to phylotypes IA-1 or II, suggesting a high degree of similarity among the strains. Because *C. acne*s primarily interacts with epithelial cells on the skin surface or sebocyte cells in sebaceous glands (Sanford et al., 2019, 2016), further analysis focused on these cell types.

### *C. acnes* sequence type is associated with acne lesions but does not predict the capacity to induce cytokine release by sebocytes

**Figure 2.**
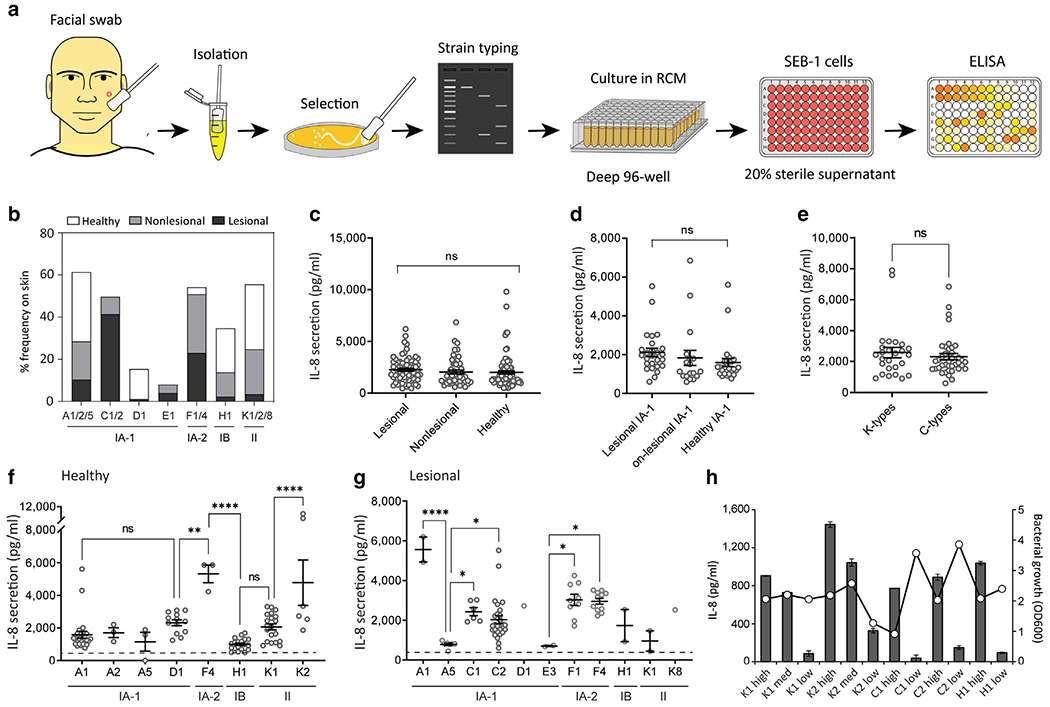
High-throughput typing and functional screening of *C. acnes* isolates from acne and healthy-derived skin sites. (**a**) Schematic for the high-throughput *C. acnes* screen, including strain collection, isolation, selection, typing, and functional readout by ELISA. (**b**) Percentage (%) frequency of the *C. acnes* phylotypes and sequence types identified from swabs of lesional and nonlesional acne skin, including healthy control skin. (**c**–**e**) IL-8 cytokine release in sebocyte SEB-1 cells 24 h after treatment with 15% sterile supernatant of *C. acnes* isolates distinguished by (**c**) source site, (**d**) phylotype IA-1, and (**e**) SLST. (**f, g**) IL8 release in SEB-1 cells 24 h after treatment with 15% sterile supernatant of all typed *C. acnes* isolates recovered from (**f**) healthy skin swabs and (**g**) lesional acne skin swabs. (**h**) Validation and designation of *C. acnes* strains based on inflammatory potential (low, medium, and high) and IL-8 release in SEB-1 sebocytes. Optical density (OD600) readings of the *C. acnes* cultures used are indicated on the secondary y-axis. n = 3. Data shown indicate mean ± SEM; *P < 0.05, **P < 0.01, ***P < 0.001, and ****P < 0.0001 with ordinary one-way ANOVA with Tukey’s multiple comparison test. Each spot represents a single isolate. med denotes medium. h, hour; ns, not significant; RCM, reinforced clostridial media; SLST, single-locus sequence type.

We next generated a larger repository of *C. acnes* isolates (>1,000) collected from swabs of facial skin sites of patients with acne as well as the facial skin of healthy volunteers (Figure 2a). Surface swabbing is a convenient method to sample the acne microbiome and provides diversity profiles similar to those of extracted follicular casts (Hall et al., 2018). Swabs were collected from both lesional and adjacent nonlesional sites of 12 subjects with acne to explore the local diversity of *C. acnes* and compare it with that of healthy skin sites of eight subjects without acne. Next, 219 *C. acnes* isolates were selected on the basis of optical density readings (OD600) >1.0 and were strain typed using single-locus sequence type (SLST). A high frequency of phylotype IA-1 C-type was observed on acne lesional skin, whereas only a few were detected on non-lesional skin, and none were recovered from healthy skin (Figure 2b). In contrast, phylotype II K-type strains were strongly associated with healthy and nonlesional skin but rarely detected on lesional skin sites. Although prior reports suggested that specific clonal complexes (CCs) of phylotype IA-1 (namely CC1, CC3, and CC4) were predominantly associated with acne (McDowell et al., 2012), our observations expand upon this finding to show that phylotype IA1 CC3 types (C1/C2 by SLST) are dominant on acne lesional sites and are rarely detected on nonlesional skin of subjects with acne.

Next, the inflammatory capacity of individual *C. acnes* isolates was measured by assessment of IL-8 secretion. The SEB-1 sebocyte cell line was selected for screening because these cells respond similarly to NHEKs (Sanford et al., 2019) but show less batch-to-batch variability than primary cell cultures and are thus more suitable for high-throughput screening. No significant difference in IL-8 release was observed on the basis of the site of recovery, disease association, or comparing C-types with K-types (Figure 2c–e). However, further resolution of additional sequence types (A, C, D, E, F, H, or K) showed significant differences in IL-8 release between strains isolated from both healthy subjects and lesions from subjects with acne (Figure 2f and g). Furthermore, substantial variation in IL-8 release was observed for isolates within the same sequence type. This suggested that important functional differences exist within similar sequence types. To assess these at greater depth, 12 isolates belonging to K1, K2, C1, C2, and H1 types were selected, validated, and named according to their sequence type and capacity to induce low, medium, or high cytokine responses in SEB-1 cells (Figure 2h). Variation in potential growth density was observed within this group but this did not correlate with differences in the capacity to promote IL-8 release (Figure 2h).

### A linear plasmid in *C. acnes* is associated with an increased capacity to induce IL8

**Figure 3.**
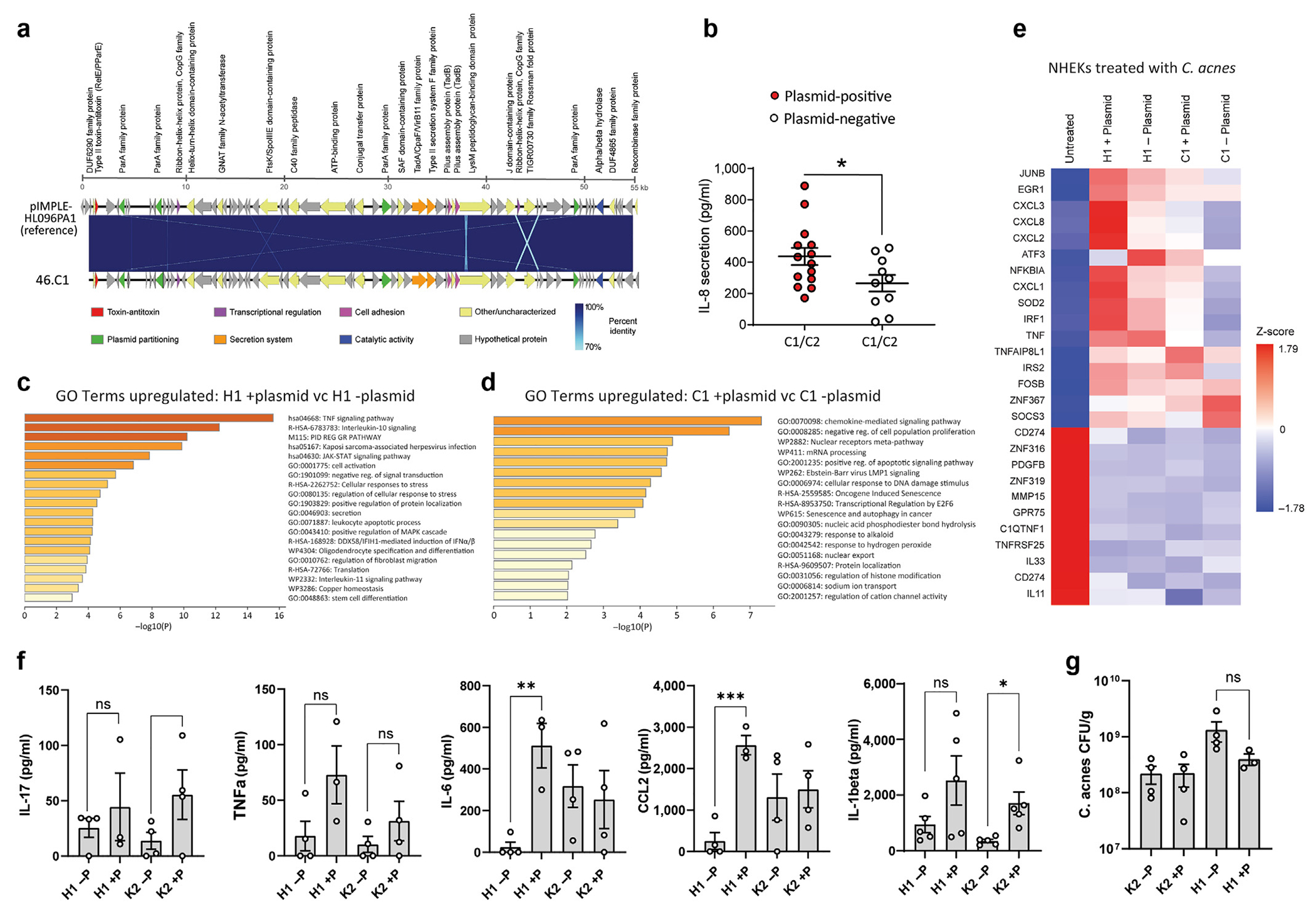
*C. acnes* plasmid-positive strains promote greater inflammation in vitro and in vivo. (**a**) *C. acnes* reference plasmid (NCBI RefSeq: NC_021086.1) from the strain pIMPLE-HL096PA1 aligned and mapped to *C. acnes* plasmid from strain 46.C1. The 71 coding sequences from both plasmid genomes are illustrated by arrows pointing toward their respective orientation. Protein-coding regions are annotated on the basis of known information from UniProt prokaryotic protein database. Blue lines within alignment indicate percentage identity. Sequences are 99.3% identical. The figure was visualized by (**b**) EasyFig 2.2.2. SLST C1-type and C2-type *C. acnes* strains were screened for the presence or absence of the linear plasmid on the basis of PCR amplification of the plasmid-borne tight adhesion (tadA) locus. SEB-1 cells were treated for 24 h with 20% sterile supernatant from plasmid-positive (+) and plasmid-negative (−) strains, and IL-8 release was measured by ELISA. (**c**) List of significantly different GO terms upregulated after treatment with H1 plasmid (+) versus H1 plasmid (−) strains. (**d**) List of significantly different GO terms upregulated after treatment with C1 plasmid (+) versus C1 plasmid (−) strains. (**e**) Heatmap expression of selected genes within the cytokine-mediated signaling GO pathway from NHEKs 4 h after treatment with 15% RCM (untreated) or 15% sterile supernatant of plasmid-positive (+) and plasmid-negative (−) strains of *C. acnes* H1 or C1 sequence types. (**f**) ELISA of tissue proteins recovered from 8 mm biopsies of mouse back skin after injection of stationary phase *C. acnes* strains. (**g**) CFU counts of the *C. acnes* bacteria recovered from biopsies of mouse back skin 72 h after injection and normalized to the weight (grams) of the biopsy tissue. n = 3–4. Data shown indicate mean ± SEM; *P < 0.05 and **P < 0.01, with two-tailed paired Student’s *t*-test. CFU, colony-forming unit; GO, gene ontology; h, hour; NCBI RefSeq, National Center for Biotechnology Information reference sequence; NHEK, normal human epidermal keratinocyte; ns, not significant; RCM, reinforced clostridial media; SLST, single-locus sequence type.

The panel of 12 strains was next subjected to whole-genome sequencing to search for the presence of genetic elements associated with inflammation. Analysis of the core genome of the identical sequence types revealed an expected high degree of gene conservation between strains. However, several strains contained distinct contigs with a GC content of (62–63%), which is approximately 2–3% higher than the chromosomal genome, suggesting the presence of a plasmid with similarity to a 56 kb linear plasmid belonging to type IA strain called pIMPLE-HL096PA1 (Figure 3a) (Davidsson et al., 2017). We identified the presence of this plasmid in sequence contigs from high inflammatory strains (35K2 high, 46C1 high, 61C2 high, and 44H1 high) and found protein-encoded genes in these plasmids with high sequence similarity to the annotated pIMPLE plasmid (Supplementary Figure S1). In contrast, none of the low- or medium-inflammatory strains in the K-, C-, or H-type strains contained the plasmid. To better assess a potential link for the presence of the plasmid with the release of IL-8, we PCR screened and identified additional plasmid-positive and plasmid-negative strains on the basis of the presence or absence of the plasmid-encoded tight adhesion gene (*tadA*) (Davidsson et al., 2017). Overall, we detected a significant increase in IL-8 from several but not all plasmid-positive strains compared with that from strains that lacked a plasmid (Figure 3b).

Having defined *C. acnes* strains with high- and low-inflammatory potential, we next sought to better understand the global transcriptional response of the host to these strains. NHEKs were treated for 4 hours with control bacterial culture media that was not conditioned by *C. acnes* (untreated) or with sterile conditioned medium from different strains of *C. acnes* (44H1 + plasmid [high], 17H1 – plasmid [low], 46C1 + plasmid [high], and 42C1 – plasmid [low]). NHEK transcriptomes were analyzed by bulk RNA sequencing. Gene ontology–based overrepresentation analysis of NHEKs treated with both H1 types revealed significant enrichment of genes relating to TNF, IL-10, and Jak–signal transducer and activator of transcription signaling pathways for the plasmid-containing strain only (Figure 3c). Likewise, in NHEKs treated with C1-type strains, the presence of the plasmid was associated with greater enrichment of genes associated with chemokine-mediated signaling and cellular response to DNA damage stimulus (Figure 3d). Examination of the expression of selected genes in the cytokine-mediated signaling gene ontology term across all treatments revealed greater expression of chemokines CXCL1, CXCL2, CXCL3, and CXCL8 for the H1 and C1 plasmid-containing strains than for their plasmid-absent counterparts (Figure 3e). These observations supported prior ELISA-based screening that suggested that strains containing the linear plasmid had greater proinflammatory potential.

### Plasmid-positive strains promote greater tissue inflammation in mouse skin

Because the capacity of *C. acnes* to promote greater inflammation in keratinocytes (KCs) and sebocytes was associated with the presence of the plasmid, we examined whether the plasmid also drives greater tissue inflammation. SKH1 mice were intradermally injected with four strains of *C. acnes* representing H1 and K2 sequence types with (+P) or without (−P) the plasmid. Seventy-two hours after infection, biopsies of the infected skin lesions were collected and cytokine levels were quantified by ELISA. The total number of *C. acnes* by CFUs was also measured. Both the 44H1 and 35K2 plasmid-containing strains promoted greater tissue inflammation than their identical nonplasmid-containing sequence types. Both IL-6 and CCL2 were significantly increased for the 44H1 +P strain compared with that for 17H1 −P strain, and IL-1β was significantly enhanced for the K2 +P strain compared with that for K2 −P (Figure 3f). The recorded increase in inflammation by H1 and K2 +P strains was unlikely to be due to differences in bacterial survival or proliferation in the tissue because total CFU was not significantly different (Figure 3g). This suggested a differential host immune response toward bacteria during dermal infection and validated previous data of inflammatory reactions to +P strains by NHEK and SEB-1 cells.

### Single-cell RNA sequencing of *C. acnes*–infected mice reveals unique inflammatory fibroblast subsets associated with the linear plasmid

**Figure 4.**
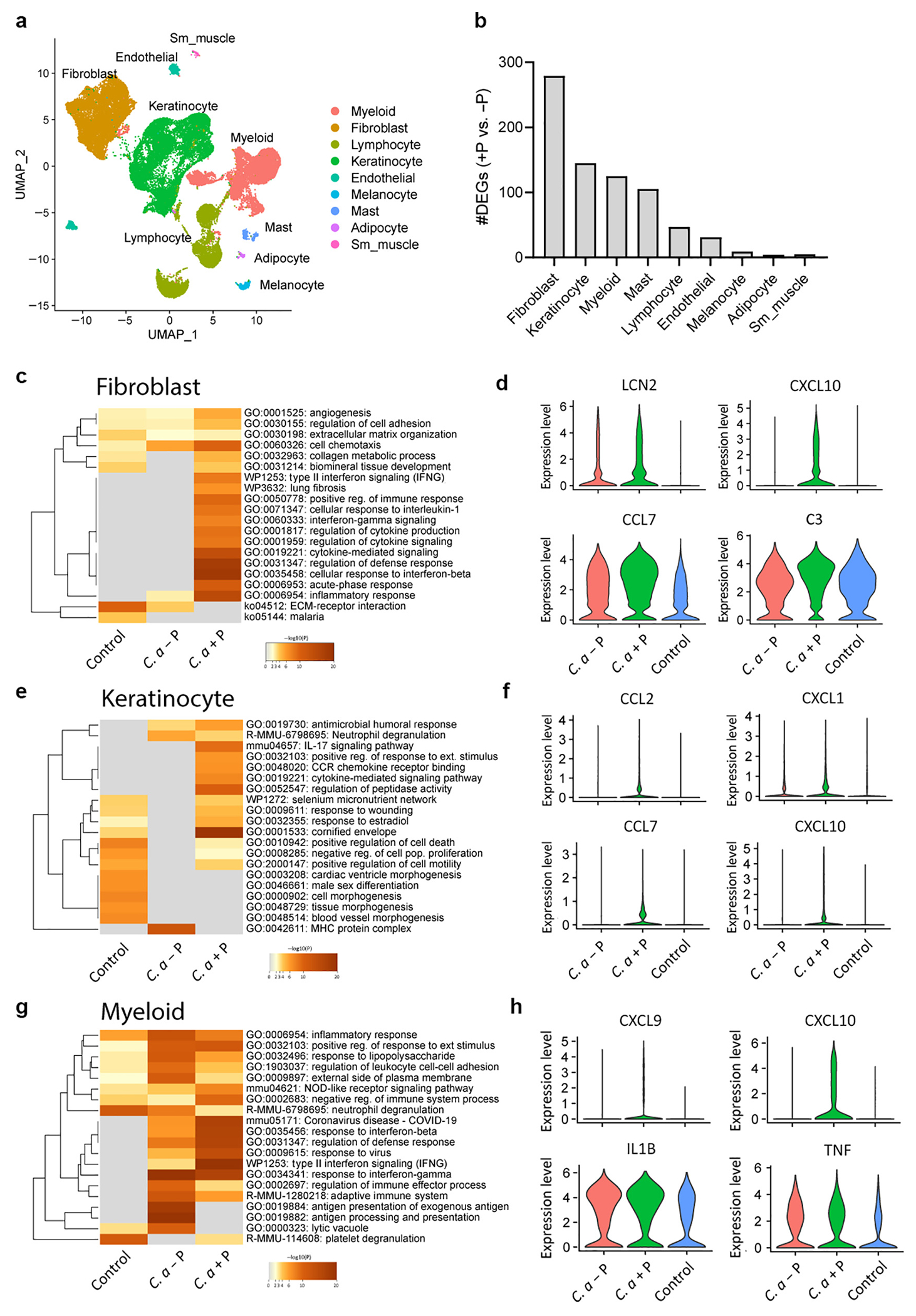
scRNA-seq reveals immune activation of distinct cell types associated with the presence of the plasmid. scRNA-seq was conducted on skin lesions 3 days after *C. acnes* infection or on mock-infected control skin of SKH-1 mice. (**a**) UMAP plot of all cells passing initial QC, showing cell-type assignment on the basis of established lineage markers. (**b**) Bar chart of the number of DEGs in each major cell type for +P *C. acnes* versus −P C. acnes. (**c, e, g**) Heatmaps of selected GO terms in (**c**) fibroblasts, (**e**) keratinocytes, and (**g**) myeloid cells enriched during *C. acnes* infection or RCM control mock infection. (**d, f, h**) Violin plots of the expression levels of four selected immune-related genes that had greater enrichment in +P *C. acnes* versus −P *C. acnes* in (**d**) fibroblasts, (**f**) keratinocytes, and (**h**) myeloid cells. n = 3 pooled biopsies of three mice. +P denotes with plasmid, and −P denotes without plasmid. DEG, differentially expressed gene; GO, gene ontology; QC, quality control; RCM, reinforced clostridial media; scRNA-seq, single-cell RNA sequencing; UMAP, Uniform Manifold Proximation and Projection.

Single-cell RNA sequencing of *C. acnes*–infected mouse skin was next performed to identify the specific cell types that mediate the differential skin immune response to the plasmid-positive strains of *C. acnes*. SKH1 mice were injected with two C-type +P and −P *C. acnes* strains, and the transcriptome for each major cell type was measured. Nine major cell types were identified and visualized using Uniform Manifold Proximation and Projection (Figure 4a). The *C. acnes* plasmid-containing strain (+P) showed greater changes to the transcriptome than the plasmid-negative strains (−P), as demonstrated by the greater number of differentially expressed genes across all nine major cell types (Figure 4b). Fibroblasts were the major cell types that responded to *C. acnes* infections, followed by KCs and myeloid cells. Gene ontology term analysis revealed that the *C. acnes* +P strain promoted greater immune responses in fibroblasts (Figure 4c and d), KCs (Figure 4e and f), and myeloid cells (Figure 4g and h) than the −P strain, including gene ontology networks relating to cytokine-mediated signaling;pathways associated with IL-1, IFN-γ, and IFN-β responses;and IL-17 signaling. In fibroblasts, major differentially expressed immune-related genes included *Cxcl10, Cccl7*, and *C3* (Figure 4d), which participate in anti-infectious immunity by immune cell recruitment during infection (Caetano et al., 2023). In addition, expression of the antimicrobial peptide–encoding gene *Lcn2* was higher in +P-activated fibroblasts and was previously found to be induced in the lesional skin of patients with acne and in response to intradermal injection of *C. acnes* in mice (Kelhälä et al., 2014; O’Neill et al., 2022). Overall, single-cell analysis of whole mouse tissue revealed greater inflammatory and host defense activation across several major cell types in response to *C. acnes* that harbor a linear plasmid.

## DISCUSSION

In this study, we demonstrate that *C. acnes* isolates representing the same sequence type exhibit distinct inflammatory responses in host cells. Our findings show that *C. acnes* sequence types previously thought to be associated with the individual affected with acne do not reflect the disease susceptibility of the individual but rather with the presence or absence of a lesion at the collection site. Furthermore, whole-genome sequencing of a panel of high- and low-inflammatory strains recovered from both acne lesions and normal skin revealed a linear plasmid associated with greater inflammation in vitro and in vivo. Transcriptional analysis of human cells and mouse tissue exposed to *C. acnes* strains containing this plasmid revealed induction of important inflammatory pathways relating to cytokine- and chemokine-mediated signaling and responses to IL-1. These data indicate that subtle differences in the genetic and metabolic diversity of identical sequence types of *C. acnes* can induce markedly different inflammatory outcomes, suggesting some redundancy defining strain functionality exclusively on disease association or sequence type alone.

*C. acnes* contains a linear plasmid initially identified by Kasimatis et al. (2013). It is described as a low copy, linear plasmid of 56 kb in size and contains a gene locus for tight adherence that codes for the biosynthesis of adhesive fimbrial low-molecular-weight protein pili, predicted to be involved in attachment. A previous study identified several plasmid-positive strains among different SLSTs indicating that plasmid carriage is not strictly associated with a single phylotype or disease type (Davidsson et al., 2017). However, no study has yet demonstrated whether the plasmid is associated with inflammation. The linear plasmid has been shown to be functionally active, with several plasmid-encoded proteins detected in secreted protein fractions of *C. acnes* (Holland et al., 2010). However, many plasmid-encoded genes have an unknown function and have yet to be characterized. Many plasmid-encoded proteins (provided in Supplementary Figure S1) showed a similar sequence similarity between 46.C1, 61.C2, and 44H1 strains but not for the 35K2 strain. Attempts to cure the plasmid from *C. acnes* strains using treatment with acridine orange and shifts to elevated temperature were attempted in this study but ultimately unsuccessful. Other groups have reported the unstable nature of the plasmid in *C. acnes* (Scholz et al., 2016), and similarly, attempts to purify and concentrate the linear plasmid were also unsuccessful, potentially owing to its reported low copy number (2–3 copies per cell) (Kasimatis et al., 2013).

Alternatively, intradermal injection of *C. acnes* into mouse skin revealed significantly greater inflammation for the plasmid-containing strain, with greater production of CCL2, IL-6, and IL-1β, than for the nonplasmid-containing strain. Single-cell RNA sequencing revealed that the major cell type contributing to immune activation in response to the *C. acnes* plasmid was dermal fibroblasts. KCs also demonstrated a greater number of differentially expressed genes in response to the *C. acnes* plasmid, as well as myeloid cells, but to a much lesser extent. That fibroblasts showed greater immune activation than other skin cell types during infection with the *C. acnes* +P strain was not entirely unsuspected given the intradermal injection site and transient 3-day post analyses in this mouse model. A topical colonization model could be adopted to better determine KC responses more accurately to *C. acnes* but, to our knowledge, has not been described in the literature. Nevertheless, recent work has shown that *C. acnes* activates an adipogenic and host defense program in fibroblasts during acne development, which is a major contributor to the pathophysiology of this disease (O’Neill et al., 2022).

In summary, these results provide greater insight into the genetic variables present in *C. acnes* that can trigger skin inflammation. Our findings demonstrate that individual colonization by a *C. acnes* sequence type does not predict susceptibility to disease. Although several factors produced by *C. acnes* can influence inflammatory potential, this study identifies an additional variable that can contribute to this complex disease. Further bacterial transcriptomic and proteomic analysis as well as technological advances to improve the capacity to perform genetic modifications in *C. acnes* are required to identify the mechanism by which a plasmid may promote inflammatory potential. Acne is a disease of the holobiome (Bordenstein and Theis, 2015), dependent on variables present in both the host and microbiome, and future progress requires coordinated study of both microbe and human cells.

## MATERIALS AND METHODS

### Human subjects and skin swab collection

All experiments involving human subjects were carried out according to protocols approved by the University of California San Diego (La Jolla, CA) institutional review board (project number 140144). Written informed consent was obtained from all subjects. *C. acnes* clinical isolates were obtained by swabbing lesional and nonlesional facial skin sites of 12 patients with mild–moderate acne as well as facial skin from 8 healthy volunteers. Patients had no history of antibiotic use within 2 weeks prior to sampling. Nonlesional skin refers to noninflamed skin sites that were directly adjacent to the lesional inflamed skin site. Subjects included both males and females aged 18–24 years, with patients with acne diagnosed with mild-to-moderate facial acne. Patients with acne at the time of sample collection were instructed to avoid any washing and cosmetic application to the skin sites in the preceding 24 hours. Healthy subjects also had no history of antibiotic use within 2 weeks prior to sampling nor had any history of inflammatory acne. For *C. acnes* strain collection, Puritan foam-tipped swabs were first soaked in sterile saline solution and applied directly to the lesional, nonlesional, and healthy skin sites. The swabs were then transferred to Eppendorf tubes containing 20% glycerol in reinforced clostridial media (RCM). The swabs were incubated for 5 minutes in each tube at room temperature with intermittent vortexing. Swabs were then transferred to −80 °C until plating. Upon thawing, the tubes were vortexed once more, and 100 μl was removed and serially diluted onto brucella blood agar plates supplemented with vitamin K, hemin, and 5% sheep’s blood and incubated for 5 days at 37 °C in an AnaeroPak (Thermo Fisher Scientific, Waltham, MA).

### Bacterial strains and bacterial culture

*C. acnes* strains were grown on Brucella blood agar plates and grown for 5 days at 37 °C under anaerobic conditions. Single colony isolates were resuspended in 5 ml RCM (BD Difco, BD, Franklin Lakes, NJ) and grown for 5–7 days at 37 °C under anaerobic conditions. For cellular bacterial supernatant treatment, bacteria were cultured in EpiLife cell culture medium (Thermo Fisher Scientific) supplemented with RCM and 1% glycerol as a lipid source for 5 days (Sanford et al., 2019). Because undiluted RCM can exhibit some inflammatory activity, the bacterial conditioned supernatant was sterile filtered and inoculated onto cells at a final concentration of 15–20%, where indicated. For in vivo and antimicrobial experiments, bacteria were pelleted and resuspended and diluted in fresh RCM to approximately 1 × 10^7^ CFU. To examine the effects of *C. acnes* in vitro, supernatants were filtered through a 0.22 μm filter. The sterile filtrate was then added to cultured cells. Of the sequenced *C. acnes* strains, the following were isolated: 21.K2low, 35.K2high, and 48.K1med for healthy skin; 2.K1low, 21.K2low, 44.H1high, and 61.C2high for acne nonlesional skin; and 5.C2low, 17.H1low, 42.C1low, 46.C1high, and 54.K1high for acne lesional skin.

### Functional screen of *C. acnes* clinical isolates

For the *C. acnes* strain collection, facial swabs were incubated in RCM, vortexed, and plated onto brucella blood agar plates supplemented with vitamin K, hemin, and 5% sheep’s blood and incubated for 5 days at 37 °C in an AnaeroPak (Thermo Fisher Scientific). The resulting small, white colonies that grew were indicative of *Cutibacteria* species. Roughly 30–50 colonies per patient were picked using a sterile inoculating loop and transferred to a single well of a deep 96-well plate containing 1 ml of RCM. After 5 days of anaerobic culture, the wells that resulted in visible, turbid growth were selected for screening and transferred to a new deep 96-well plate containing 1 ml of EpiLife supplemented with 50% RCM and 1% glycerol. Plates were cultured for 5 days, and strains that grew to at least optical density 1.0 (optical density of 600 nm) were selected for screening. To functionally screen for inflammatory potential, half of the culture supernatant was transferred to a 96-well 0.2 μm filter plate and centrifuged at 4,000*g* for 15 minutes. Sterile glycerol was added to the remaining bacterial culture (at a final concentration of 20%), and the plates were frozen at −80 °C for long-term storage. Next, 20% sterile culture filtrate was added to SEB-1 cells for 24 hours. The supernatants of the SEB-1 cells were then further filtered and transferred to IL-8 ELISA plates (R&D Systems, Minneapolis, MN) for quantification.

### SLST

Putative *C. acnes* colonies cultured on blood agar 5–7 days after anaerobic growth were distinguished from other nonspecific colonies, such as *Staphylococci*, on the basis of size, hemolysis, color, morphology, and other morphological properties. Between 15 and 20 CFUs were picked per patient for SLST. Colonies were picked by sterile toothpick and inoculated into PCR-grade water. A volume of this bacterial suspension was used to perform a colony PCR according to Scholz et al. (2014). The resulting single loci amplicons were subject to PCR purification and submitted for Sanger sequencing. The resulting sequences were submitted to the online *C. acnes* SLST database (http://medbac.dk/slst/pacnes) to determine the *C. acnes* sequence type.

### Animals and animal care

All animal experiments were approved by the University of California San Diego Institutional Animal Care and Use Committee (protocol number S09074). SKH-1 hairless mice and wild-type, *Tlr2*^−/−^ C57/Bl6 mice were originally purchased from The Jackson Laboratory (Bar Harbor, ME), bred, and maintained in the animal facility of the University of California San Diego. Animals in all experimental models were age and sex matched.

### Mouse model of *C. acnes* skin infection

To promote the formation of acne-like lesions in mice, 100 μl of squalene was topically applied to the backs of age-matched (8–10 weeks) SKH-1 mice 24 hours before infection and every 24 hours thereafter throughout the duration of the experiment according to (O’Neill et al. (2022)).

### RNA isolation, cDNA synthesis, and RT-qPCR analysis

Cultured cells and isolated tissues with lysed PureLink Lysis Buffer (Ambion/Life Technologies, Carlsbad, CA) and RNA were isolated using the PureLink isolation kit. Up to 1 μg of RNA was reverse transcribed to cDNA using the Verso cDNA synthesis kit (Thermo Fisher Scientific). RT-qPCR was performed in the CFX96 Real-Time System (Bio-Rad Laboratories, Hercules, CA) using SYBR Green Mix (Biomake, Houston, TX). The housekeeping gene *GAPDH* was used to normalize gene expression in samples.

### Single-cell RNA-sequencing analysis

For single-cell RNA-sequencing analysis of mouse skin, the upper back skin of SKH-1 mice was intradermally infected with 1 × 10^7^ CFU of *C. acnes* or RCM control for 3 days. Skin samples were collected from 6 mm punch biopsies and immediately placed on ice. In total, three biopsies were pooled per condition (infected or noninfected) for single-cell generation. Isolation and analyses of viable single cells were as previously described (O’Neill et al., 2022).

### Statistical analysis

Statistical significance was calculated using a one-way ANOVA with Tukey’s multiple comparison test and Student’s two-tailed *t*-test where indicated (*P < 0.05, **P < 0.01, ***P < 0.001, and ****P < 0.0001 as indicated in figure legends).

## Supplementary Material

1

## Data Availability

All data are available in the paper or the supplementary materials. Materials will be made available by contacting RLG and at completion of a material transfer agreement. Single-cell RNA-sequencing data are available for download at gene expression omnibus GSE211279, and bulk RNA-sequencing data are available on the Dryad Server at https://doi.org/10.6076/D1488Z.

## Abbreviations

CC: clonal complex
CFU: colony-forming unit
KC: keratinocyte
NHEK: normal human epidermal keratinocyte
RCM: reinforced clostridial media
SLST: single-locus sequence type
