# Supplementary material for: Genetic and Functional Analyses of Cutibacterium Acnes Isolates Reveal the Association of a Linear Plasmid with Skin Inflammation
Source: J Invest Dermatol. Author manuscript; Available in PMC 2025 Jan 1. (PMC11137742; doi:10.1016/j.jid.2023.05.029)
Supplement: 1 [file NIHMS1993267-supplement-1.pdf]

SUPPLEMENTARY MATERIALS AND METHODS

Cell culture

Normal neonatal human primary epidermal keratinocytes were cultured in Epilife complete medium containing 60 mM calcium chloride supplemented with 1X human keratinocyte growth supplement and a 1X antibiotic-antimycotic at 37 °C and 5% carbon dioxide. Normal neonatal human primary epidermal keratinocytes were used only for experiments between passages 3 and 5. Normal neonatal human primary epidermal keratinocytes were grown to approximately 80% confluency prior to experimentation. Human monocyte-derived dendritic cells were isolated from human PBMCs prepared by centrifugation on a Ficoll gradient. Blood CD14+ monocytes were isolated from PBMCs by positive selection using anti-CD14−coated magnetic beads according to the manufacturer's instructions (Miltenyi Biotek, Bergisch Gladbach, Germany). The adherent monocytes were cultured in DMEM supplemented with 10% fetal bovine serum supplemented with 600 U/ml GM-CSF and 20 ng/ml IL-4. On day 4, the medium was replenished containing GM-CSF, IL-4, and TNF-α (final concentration of 10 ng/ml). After 7 days of culture, the cells were washed and replaced with DMEM/fetal calf serum. Human monocyte-derived dendritic cells were serum starved for 4 hours prior to experimentation and for the duration of the treatment. For bacterial supernatant treatments, differentiated normal neonatal human primary epidermal keratinocytes and human monocyte-derived dendritic cells were treated with sterile-filtered bacterial supernatant at 15−20% (volume) in Epilife medium for 4 hours or 16−24 hours (as indicated). Sebocyte SEB-1 cells were cultured in and maintained in Sebomed Basal Medium supplemented with 10% fetal bovine serum and recombinant human epidermal GF (5 ng/ml) at 37 °C in 5% carbon dioxide. Cells were passaged before reaching 100% confluency in tissue culture flasks. For experiments, cells were switched to medium containing 1% fetal bovine serum overnight before stimulation. For RNA extraction, the cells were washed twice in PBS and lysed with PureLink lysing Buffer supplemented with β-mercaptoethanol. RNA was purified according to the manufacturer's instructions.

Reagents and chemicals

Recombinant IL-4 (10 μg), recombinant TNF-α (10 μg), recombinant IFN-γ (10 μg), and lipopolysaccharide were purchased from BioLegend (San Diego, CA). Anaerogen sachets (OXAN0025A) were purchased from Thermo Fisher Scientific (Waltham, MA). GasPak EZ (B260683) was purchased from Thermo Fisher Scientific. MALP-2 was purchased from Enzo Life Sciences (Farmingdale, NY). Synthetic LL-37 was purchased from Genemed Synthesis (San Antonio, TX). Lauric acid (W261416) was purchased from Sigma-Aldrich (St. Louis, MO). Synthetic cathelicidin-related antimicrobial peptide was purchased from Sigma-Genosys (Woodlands, TX). IL-8 ELISA (BDB555244) and IL-6 ELISA (BDB555220) were purchased from Thermo Fisher Scientific. IL-17, IL-10, CCL2, and TNF-α ELISA Duoset were purchased from Thermo Fisher Scientific. Complete protease inhibitor cocktail tablets (11697498001) were purchased from Thermo Fisher Scientific.

Mouse model of *Cutibacterium acnes* skin infection

To promote the formation of acne-like lesions in mice, 100 μl of squalene was topically applied to the backs of age-matched (8−10 weeks) SKH-1 mice 24 hours before infection and every 24 hours thereafter throughout the duration of the experiment according to O'Neill et al. (2022). Briefly, mice were intradermally injected with approximately 1 × 107 colony-forming units of *C. acnes* or control (reinforced clostridial media). Images of the mouse back skin were taken with a Panasonic Lumix TS30 digital camera. Three days after injection, mice were killed, and an 8-mm skin biopsy of the infected region was retrieved. For protein extraction, skin samples were cut into small pieces using a sterile scalpel and then digested in RIPA buffer supplemented with protease inhibitors for 60 minutes on ice, vortexing intermittently every 5 minutes. Tubes were centrifuged at 10,000*g* for 10 minutes at 4 °C and the supernatant was harvested. Protein concentration was quantified by Pierce BCA assay kit and normalized to 500 μg/ml. Selected cytokines were quantified by ELISA according to the manufacturer's instructions.

Single-cell RNA-sequencing analysis

The 10X Genomics (Pleasanton, CA) Cell Ranger (version 3.0.1) software pipeline with default parameters was used to perform sample demultiplexing, barcode processing, alignment to the mm10 reference genome, and single-cell gene counting. Data were further filtered, processed, and analyzed using the Seurat R toolkit, version 3 (Butler et al., 2018; Stuart et al., 2019). Filtering of initial data involved selecting cells with >100 features and <10% mitochondrial genes. Data were normalized and scaled, and variable genes were identified using the function scTransform(a). Principal components were calculated from these variable genes using RunPCA(a), and the top 30 principal components were used for downstream analysis. Clusters were identified using FindNeighbors(a) and then FindClusters(a)with argument resolution = 2.0. Nonlinear dimensionality reduction and visualization were performed with Uniform Manifold Proximation and Projection using the RunUMAP(a) function. Marker genes for each cluster were determined using FindAllMarkers(a) with parameters only.pos = TRUE, min.pct = 0.25, and thresh.use = 0.25. Clusters were assigned to cell types myeloid, fibroblast, lymphocyte, keratinocyte, endothelial, melanocyte, mast, adipocyte, and smooth muscle on the basis of marker genes. A total of 14 of the original 53 clusters were annotated as fibroblasts and underwent additional quality control. In brief, a new Seurat object was generated using only the cells in these clusters with their corresponding raw counts, followed by normalization, scaling, clustering, and dimensionality reduction as described earlier, resulting in 15 fibroblast clusters with FindClusters(a) resolution argument = 0.6. Clusters with high expression of leukocyte, keratinocyte, and adipocyte marker genes as well as cell cycle genes were excluded. A final Seurat object was created from the remaining cells and their raw counts with a final iteration of normalization, scaling, clustering, and dimensionality reduction, resulting in 12 fibroblast clusters with FindClusters(a) resolution argument = 0.6. Marker genes were identified in the three groups with FindAllMarkers(a) with parameter only.pos = TRUE and

thresh.use = 0.25. Gene ontology analysis was performed on marker genes using Metascape (Zhou et al., 2019).

Whole-genome sequencing and plasmid assembly

Overnight cultures of 12 *C. acnes* strains were pelleted by centrifugation at 10,000*g* for 5 minutes and transferred to a −80 °C freezer. The bacterial pellets were shipped to The Sequencing Center (Fort Collins, CO) for genomic DNA extraction, library preparation, and Illumina short-read sequencing. First, sequencing adapters were trimmed using Trimmomatic, version 0.39 (Bolger et al., 2014). Second, overlapped reads were merged using FLASH, version 1.2.11 (Magoč and Salzberg, 2011), to support the generation of longer reads from fragment libraries before genome assembly. Third, reads were assembled using Spades, version 3.12.0, with the following parameters: –merge to indicate merged forward and reverse paired-end reads and –plasmid to initiate the plasmidSPAdes algorithm for plasmid-specific detection and assembly (Bankevich et al., 2012). A fully assembled plasmid mapped onto one contig was achieved for *C. acnes* strain 46.C1. This plasmid sequence was annotated using Prokka, version 1.12, with default parameters to obtain the location and translation of each coding sequence (CDS), and the putative proteins were identified through the Uniprot prokaryotic database. Next, the *C. acnes* reference plasmid was mapped to the *C. acnes* plasmid from strain 46.C1 using EasyFig 2.2.2 default parameters to show percentage identity across the genome and the 71 coding sequences (Figure 3a). Gray arrow annotations indicate hypothetical proteins. The 25 nonhypothetical proteins' recognized names from the National Center for Biotechnology Information reference are reported respectively above their genome annotation. Other colored arrows indicate whether the nonhypothetical protein is present in the 46.C1 plasmid and any known information on protein functionality according to UniProt.

Plasmid sequences from all *C. acnes* strains (46.C1, 61.C1, 44.H1, and 35.K2) were each independently compared with the *C. acnes* reference plasmid pIMPLE-HL096PA1 (National Center for Biotechnology Information reference sequence: NC_021086.1). First, all plasmid sequences of interest were annotated using Prokka, version 1.12, as mentioned previously. Next, using an in-house Python script, Biopython-SeqIO, version 1.79, was used to parse the GenBank files for CDS features from each annotated plasmid of interest. For each of the four plasmids in this study, the 71 CDS regions from pIMPLE-HL096PA1 were independently aligned to every CDS region to identify the analogous or highest similarity region (Supplementary Figure S1). Percentage identities were calculated from Levenshtein (edit) distance using Python-Levenshtein, version 0.12.2. The highest percentage match, if ≥60%, was reported, with a value of 1 indicating a 100% sequence match of a CDS region between the reference and the plasmid of interest.

SUPPLEMENTARY REFERENCES

Bankevich A, Nurk S, Antipov D, Gurevich AA, Dvorkin M, Kulikov AS, et al. SPAdes: a new genome assembly algorithm and its applications to single-cell sequencing. J Comput Biol 2012;19:455−77.

Bolger AM, Lohse M, Usadel B. Trimmomatic: A flexible trimmer for Illumina sequence data. Bioinformatics 2014;30:2114−20.

Butler A, Hoffman P, Smibert P, Papalexi E, Satija R. Integrating single-cell transcriptomic data across different conditions, technologies, and species. Nat Biotechnol 2018;36:411−20.

Magoč T, Salzberg SL. FLASH: fast length adjustment of short reads to improve genome assemblies. Bioinformatics 2011;27:2957.

O'Neill AM, Liggins MC, Seidman JS, Do TH, Li F, Cavagnero KJ, et al. Antimicrobial production by perifollicular dermal preadipocytes is essential to the pathophysiology of acne. Sci Transl Med 2022;14: eabh1478.

Stuart T, Butler A, Hoffman P, Hafemeister C, Papalexi E, Mauck WM 3rd, et al. Comprehensive integration of single-cell data. Cell 2019;177: 1888−902.e21.

Zhou Y, Zhou B, Pache L, Chang M, Khodabakhshi AH, Tanaseichuk O, et al. Metascape provides a biologist-oriented resource for the analysis of systems-level datasets. Nat Commun 2019;10:1523.

Protein name	NCBI Accession ID	46.C1	61.C1	44.H1	35.K2
Hypothetical protein	N/A				
Hypothetical protein	N/A			1	1
Hypothetical protein	N/A				
DUF6290 family protein	WP_002520161.1	1		1	0.95
Type II toxin-antitoxin system RelE/ParE family toxin	WP_002520160.1	1		1	0.99
Hypothetical protein	N/A	0.94		0.94	
Hypothetical protein	N/A	1		1	
ParA family protein	WP_002520157.1	1		1	
Hypothetical protein	N/A				
Hypothetical protein	N/A	0.67		0.67	
Hypothetical protein	N/A	0.97		0.97	
Hypothetical protein	N/A	1		1	0.92
ParA family protein	WP_032501527.1	0.78		0.78	0.92
Hypothetical protein	N/A	1		1	0.98
Hypothetical protein	N/A	1		1	0.99
Hypothetical protein	N/A	0.99		0.99	0.97
Ribbon-helix-helix protein, CopG family	WP_002520145.1	1		1	0.89
Helix-turn-helix domain-containing protein	WP_015588734.1	1		1	0.96
Hypothetical protein	N/A	1		0.71	0.88
Hypothetical protein	N/A	1		1	0.86
GNAT family N-acetyltransferase	WP_002520142.1	1		1	0.82
Hypothetical protein	N/A	0.93		0.91	0.87
Hypothetical protein	N/A	0.75			0.73
Hypothetical protein	N/A	1			0.76
Hypothetical protein	N/A	0.92	0.9	0.92	0.83
Hypothetical protein	N/A	1	1	1	0.99
Hypothetical protein	N/A	1	1	1	0.99
Hypothetical protein	N/A	1	0.98	1	0.85
FtsK/SpoIIIE domain-containing protein	WP_002520135.1	0.91	0.91	0.91	0.88
Hypothetical protein	N/A	1	1	1	1
Hypothetical protein	N/A	1	1	1	0.99
C40 family peptidase	WP_041446720.1	1	0.95	1	0.95
Hypothetical protein	N/A	1	1	1	0.93
ATP-binding protein	WP_002520130.1	1	1	1	0.99
Hypothetical protein	N/A	1	1	1	0.94
Hypothetical protein	N/A	1	1	1	0.88
Conjugal transfer protein	WP_002520128.1	0.88	0.88	0.88	0.78
Hypothetical protein	N/A	1	1	1	0.91
ParA family protein	WP_002520127.1	0.99	0.99	0.99	0.91
Hypothetical protein	N/A	1	1	1	0.84
SAF domain-containing protein	WP_002520126.1	1	1	1	0.79

Supplementary Figure S1. Amino acid sequence comparison of the *C. acnes* reference plasmid pIMPLE-HL096PA1 to assembled plasmids from strains 46.C1, 61.C2, 44.H1, and 35.K2. 71. Coding sequences within *C. acnes* reference plasmid (NCBI Ref Seq: NC_021086.1) from the strain pIMPLE-HL096PA1 were aligned to the coding sequences within four plasmids from *C. acnes* strains 46.C1 and 61.C2, 44.H1, and 35.K2. Percentage sequence identity was calculated using the Levenshtein distance algorithm. Sequences from reference plasmid with 60% identity or higher were reported. The 25 nonhypothetical proteins are indicated in yellow. Proteins from the reference sequence sharing ≥90% identity to plasmids sequenced in this paper are indicated in green. ID, identification; N/A, not available; NCBI Ref Seq, National Center for Biotechnology Information reference sequence.

Hypothetical protein	N/A	1	1	1	0.88
TadA/CpaF/VirB11 family protein	WP_002522438.1	1	1	1	0.92
Type II secretion system F family protein	WP_002520578.1	1	1	1	0.9
Hypothetical protein	N/A	1	1	1	0.92
Hypothetical protein	N/A	1	1	1	0.84
Pilus assembly protein	WP_002519235.1	0.86	0.86	0.86	0.82
Hypothetical protein	N/A	0.87	0.87	0.87	0.85
Pilus assembly protein	WP_002519237.1	1	1	1	1
LysM peptidoglycan-binding domain-containing protein	WP_015588743.1	1	1	1	0.65
Hypothetical protein	N/A	1	1	1	0.68
Hypothetical protein	N/A	1	1	1	0.96
J domain-containing protein	WP_002520572.1	1	1	1	0.63
Ribbon-helix-helix protein, CopG family	WP_015588744.1		0.91		0.84
TIGR00730 family Rossman fold protein	WP_002522196.1	0.97	0.98	0.97	0.97
Hypothetical protein	N/A	0.88	0.88	0.88	0.88
Hypothetical protein	N/A	0.84	0.84	0.84	0.81
Hypothetical protein	N/A			1	
Hypothetical protein	N/A	1	1	1	
ParA family protein	WP_032501527.1	0.98	0.98	0.98	
Hypothetical protein	N/A	1	1	1	
Hypothetical protein	N/A	1	1	1	
Hypothetical protein	N/A	0.99	0.99	0.99	
Hypothetical protein	N/A	1		1	
Alpha/beta hydrolase	WP_002518866.1	1	1	1	
DUF4865 family protein	WP_002518867.1	1	1	1	
Hypothetical protein	N/A				
Hypothetical protein	N/A	1	1	1	
Hypothetical protein	N/A	1	1	1	
Hypothetical protein	N/A	0.86	0.86	0.97	
Recombinase family protein	WP_073859749.1			0.95	

Supplementary Figure S1. Continued.
